# Insight to changing morphologic patterns of glomerulopathy in adult Pakistani patients: an institutional perspective

**DOI:** 10.1186/s13104-016-1876-y

**Published:** 2016-02-08

**Authors:** Atif Ali Hashmi, ZubaidaFida Hussain, Muhammad Muzzammil Edhi, Shazia Mumtaz, Naveen Faridi, Mehmood Khan

**Affiliations:** Department of Histopathology, Liaquat National Hospital and Medical College, Karachi, Pakistan; Liaquat National Hospital and Medical College, Karachi, Pakistan; Dhaka Medical College, Dhaka, Bangladesh

**Keywords:** Nephrotic syndrome, Renal biopsy, Membranous glomerulonephritis, Focal segmental glomerulosclerosis

## Abstract

**Background:**

Idiopathic nephrotic syndrome encompasses diverse histogenetic patterns and depicts socioeconomic and demographic differences attributable to genetic profile, environmental factors and prevalence of infectious diseases. A lack of renal registry in our country necessitates a need to document changing histologic patterns of nephrotic syndrome as noted in different parts of the world.

**Methods:**

We retrospectively analyzed 140 patients who underwent renal biopsy at Liaquat National Hospital from January 2009 to December 2013 over a period of 3 years. On the basis of clinical profile cases were segregated into nephritic and nephrotic syndrome and histologic and immunoflourescence findings were analyzed.

**Results:**

Among 140 cases of glomerulonephritis diagnosed in the study period, 98 cases (70 %) were those of primary glomerulonephritis and 42 were of secondary glomerulopathy (30 %). Membranous glomerulonephritis was the most common primary glomerulonephritis (33.6 %) followed by focal segmental glomerulosclerosis FSGS (20.4 %), whereas lupus nephritis is the most common secondary glomerulopathy (47.6 %) followed by amyloidosis and diabetic glomerulosclerosis (16.6 % each).

**Conclusion:**

We found a considerable high incidence of membranous glomerulonephritis and FSGS in our population that entails a need to investigate prevalence of associated factors like Hepatitis B and HIV infections in population at risk. Moreover, renal biopsy registry would be instrumental in this regard to record changing disease pattern in this part of the world.

## Background

Renal diseases accredit major health problem in developing countries [1, 2]. While chronic diseases like diabetes and hypertension still accounts for the major bulk of chronic renal failure patients, idiopathic glomerulonephritis (GN) poses a major diagnostic challenge to both nephrologists and pathologists. Degree of proteinuria often helps clinicians to narrow down their differentials to two major clinical patterns: nephritic and nephrotic. While the epidemiology of nephritic syndrome changed quite a bit over the years due to changing public health protocols, prevailing infections and vaccinations, etiology of nephrotic syndrome showed more dramatic fluctuations in etiology and depicts a racial and age preference [3, 4]. Whether this represents a change in the disease pattern or better understanding of glomerular disorders is a matter of debate. However it has been suggested that apart from genetic profile, environmental factors and prevalence of infectious diseases attribute to socioeconomic and demographic differences seen in patterns of glomerulopathy. Acute nephritic syndrome is usually related to underlying immunologic injury, the culprit of which is attributed mostly to infectious agents [5, 6]. However, nephritic syndrome is usually self limited and renal biopsy is not always indicated. On the other hand nephrotic syndrome encompasses diverse histogenetic patterns ranging from minimal change disease (MCD) to collapsing form of focal segmental glomerulosclerosis (FSGS).

Renal biopsy remained the gold standard for the diagnosis of primary glomerulonephritis with ancillary immunoflorescent and electron microscopic examination. Most common indication for renal biopsy is unexplained elevation of renal parameters and proteinuria. Renal biopsy is often performed in systemic diseases like systemic lupus erythematosus when active renal involvement is suspected due to presence of hematuria or proteinuria and elevated serologic markers of lupus nephritis. In diabetes and hypertension, renal biopsy is not indicated as progressive renal involvement is usually present in uncontrolled cases of long duration disease; however it is usually done in atypical presentations to rule out other causes of glomerulopathy or de novo primary glomerulonephritis.

In addition to being different histologically, disease pattern of GN also differs significantly in clinical parameters. GN typically behaves in a self limited fashion in children, while it poses a major risk when occurring in adults. Data of adult western population show FSGS to be the most frequent pattern in African Americans while membranous GN dominates the clinical picture of nephrotic syndrome in whites. It is of immense importance to recognize common pathologic patterns of idiopathic GN in a population to devise better therapeutic protocols. Data pertaining to this clinical entity is limited in our setup, as renal biopsy registries are not currently available and changing patterns of glomerulopathy have not been well demonstrated in our setup as noted in other populations [7, 8]. Therefore we aimed to determine histopathologic patterns of both secondary and idiopathic (primary) GN in adult patients undergoing renal biopsy.

## Methods

We retrospectively analyzed 140 patients who underwent renal biopsy at Liaquat National Hospital from January 2009 to December 2013 over a period of 3 years. An approval from research and ethical review committee were taken antecedent to conducting the study. Data was retrieved by reviewing pathology request forms of renal biopsy specimens received at Histopathology laboratory during the study period. This included patient major signs and symptoms, indication of renal biopsy, laboratory findings like degree of hematuria, proteinuria, urinalysis results and positive serum markers like ANA, anti-dsDNA and c-ANCA. Cases with insufficient clinical information were excluded from the study. A total of 30 biopsies were excluded by this criterion. Moreover five cases were also excluded due to inconclusive findings on renal biopsy and two due to inconclusive immunoflourescence results. Subsequently cases were segregated according to mode of presentation and lab findings into nephrotic and nephritic syndrome. Cases with >3.5 g proteinuria with edema were defined as nephrotic syndrome and cases with <3.5 g proteinuria along with hematuria and RBC casts were classified as nephritic syndrome. General indications of renal biopsy at our institution were nephrotic or acute nephritic syndrome, unexplained rise in urea and creatinine, evaluation of extent of renal involvement in secondary disorders like SLE, DM etc. In SLE, renal biopsy was performed when there is clinical or laboratory evidence of lupus nephritis (e.g. elevated anti dsDNA levels). In diabetes and chronic hypertension, renal biopsy was only performed when some other superimposed glomerular pathology was suspected. Percutaneous renal biopsy was performed under aseptic precautions with a 16–18 gauge needle. Informed written consent was taken prior to conducting the procedure. Two separate cores were taken for light microscopy and immunoflourscence studies. Nine to 12 thin levels (2 µm) were examined on 3 to 4 slides stained with hematoxylin& eosin for light microscopy along with periodic acid shift, trichome and silver stains on each biopsy. Stains for amyloid were performed when needed. Cases with atleat six glomeruli and one artery were considered adequate. All biopsies were evaluated by two senior histopathologists trained in renal pathology. Immunofluorescence studies were performed using antibodies against IgG, IgM, IgA, C3c and C1q. Cases with provisional diagnosis of MCD or inconclusive light and immunoflourence findings were referred for electron microscopy at another institution as it was not routinely performed at our institution. Statistical analysis was performed using Chi square on SPSS version 20.

## Results

Mean age at diagnosis was 34.9 years (±16.8) with male to female ratio of 1:1.2. Age range was 20–75 years with most common age group being 31–50 years comprising of 33 % of cases. Among 140 cases of glomerulonephritis diagnosed in the study period, 98 cases (70 %) were those of primary glomerulonephritis and 42 were of secondary glomerulopathy (30 %). Membranous glomerulonephritis was the most common primary glomerulonephritis (33.6 %) followed by FSGS (20.4 %), whereas lupus nephritis is the most common secondary glomerulopathy (47.6 %) followed by amyloidosis and diabetic glomerulosclerosis (16.6 % each). There was no significant difference in sex distribution among different types of primary and secondary GN. Mean age for most forms of GN fall in third and fourth decade of life. On the other hand, the mean age for lupus nephritis, IgA nephropathy and MCD were slightly lower than other forms of GN, however the difference was not statistically significant. Frequency of primary and secondary GN alongwith age and sex distribution is shown in Tables 1 and 2.

**Table 1:** Gender distribution and mean age of primary and secondary glomerulonephritis

	Total	Male		Female		Age
		n	%	n	%	Mean
Primary glomerulonephritis						
Minimal change disease	12	7	58.3	5	41.7	30
Focal segmental glomerulonephritis	20	10	50.0	10	50.0	32
Membranous glomerulonephritis	33	17	51.5	16	48.5	38
Membranoproliferative glomerulonephritis	15	7	46.7	8	53.3	37
Acute proliferative GN	7	2	28.6	5	71.4	35
IgA nephropathy	9	4	44.4	5	55.6	27
Cresentic GN, immune complex type	2	2	100.0	0	0	44
Secondary glomerulonephritis						
Lupus nephritis	20	6	30.0	14	70.0	25
Cresentic GN, pauciimmune type/Wegener disease	3	2	66.7	1	33.3	49
Diabetic glomerulosclerosis	7	4	57.1	3	42.9	48
Amyloidosis	7	1	14.3	6	85.7	37
Hypertensive nephropathy	2	1	50.0	1	50.0	53
Chronic sclerosing glomerulonephritis (ESRD)	3	2	66.7	1	33.3	52

**Table 2:** Age distribution of primary glomerulonephritis

	Less than 18 years		19–30 years		31–50 years		More than 50 years	
	n	%	n	%	n	%	n	%
Minimal change disease	5	41.7	2	16.7	3	25.0	2	16.7
Focal segmental glomerulonephritis	3	15.0	9	45.0	5	25.0	3	15.0
Membranous glomerulonephritis	3	9.1	10	30.3	12	36.4	8	24.2
Membranoproliferative glomerulonephritis	2	13.3	4	26.7	5	33.3	4	26.7
Acute proliferative GN	2	28.6	1	14.3	2	28.6	2	28.6
IgA nephropathy	2	22.2	3	33.3	4	44.4	0	0
Cresentic GN, immune complex type	0	0	1	50.0	0	0	1	50.0

Among 98 cases of primary GN, 84 cases (85 %) presented with nephrotic syndrome. Table 3 shows the different histologic patterns of primary GN. Among nephrotic syndrome, most common pattern was membranous GN (38 %), followed by FSGS (21 %) and MPGN (16 %). On the other hand acute post streptococcal GN was the most common pattern found in nephritic syndrome patents (42 %) followed IgA nephropathy (28 %). Table 4 shows the IMF pattern of primary and secondary GN. All cases of MPGN were of type I. There was no case of goodpasture syndrome or myeloma related disorder except for amyloidosis. There were five cases of cresentic GN; out of which, two cases showed immune complex deposits on IF in the absence of significant primary GN pattern therefore they were categorized as primary GN. The rest of 3 cases were pauci-immune and they were c-ANCA positive with systemic evidence of Wegener’s syndrome, therefore they were classified as secondary GN. There were 7 cases of amyloidosis, out of which 3 were known cases of multiple myeloma, however no further stains were performed to sub classify cases of amyloidosis on renal biopsy.

**Table 3:** Clinical presentation of different types of primary glomerulonephritis

	Nephrotic syndrome		Nephritic syndrome	
	N	%	N	%
Primary glomerulonephritis				
Minimal change disease	12	14.2	0	0
Focal segmental glomerulonephritis	18	21.4	2	14.2
Membranous glomerulonephritis	32	38.0	1	7.1
Membranoproliferative glomerulonephritis	14	13.0	1	7.1
Acute proliferative GN	1	1.1	6	42.8
IgA nephropathy	5	5.9	4	28.5
Cresentic GN, immune complex type	2	2.3	0	0
Total	84		14

**Table 4:** Immunoflourescence patterns of primary and secondary glomerulonephritis

	Immunoglobulins									
	IgG		IgM		IgA		C3c		C1q	
	N	%	N	%	N	%	N	%	N	%
Primary glomerulonephritis										
Minimal change disease	2	16.6	7	58.3	4	33.3	5	41.6	2	16.6
Focal segmental glomerulonephritis	2	10.0	17	85.0	5	25.0	12	60.0	7	35.0
Membranous glomerulonephritis	28	84.8	27	81.8	18	54.5	29	87.8	23	69.6
Membranoproliferative glomerulonephritis	10	66.7	13	86.7	13	86.7	15	100	10	66.7
Acute proliferative GN	6	85.7	4	57.1	4	57.1	7	100	4	57.1
IgA nephropathy	1	11.1	9	100	9	100	8	88.9	4	44.4
Cresentic GN, immune complex type	1	50.0	2	100	2	100	2	100	2	100
Secondary glomerulonephritis										
Lupus nephritis	20	100	20	100	19	95.0	20	100	20	100
Cresentic GN, pauciimmune type/Wegener disease	1	33.3	2	66.7	0	0	2	66.7	1	33.3
Diabetic glomerulosclerosis	4	57.1	6	85.7	4	57.1	4	57.1	1	14.2
Amyloidosis	4	57.1	3	42.8	2	28.6	4	57.1	2	28.6
Hypertensive nephropathy	2	100	2	100	1	50.0	2	100	1	50.0
Chronic sclerosing glomerulonephritis (ESRD)	0	0	3	100	0	0	3	100	1	33.3

## Discussion

Our institution represents one of the largest tertiary care centers in country with well developed nephrology unit. Renal biopsy is routinely performed as part of workup in patients with unexplained deterioration of renal functions or to determine extent of renal dysfunction in some secondary renal diseases like systemic lupus erythematosus, Diabetes and hypertension. Nephrotic syndrome is the most common clinical picture in patients undergoing renal biopsies as demonstrated in our study. A survey of renal biopsies performed in United States from 1995 to 1997 showed that FSGS is the most common cause of nephrotic syndrome accounting for 35 % of all cases and more than 50 % cases among blacks [9]. An 11-fold rise in the incidence of FSGS as a cause of end stage renal disease was identified in recent year in US [10]. These changing histologic trends were also demonstrated in India. FSGS was found to be the dominant morphologic pattern in different studies. Rathi et al. found a frequency of 30 %, while Chandrika found 18 % frequency of FSGS [11, 12]. This shows a rising frequency of FSGS in some parts of the world. The statistics differ in other regions of the world. Renal biopsy registry in Spain shows membranous GN as the most common pattern (24 %), followed by MCD (6 %) and lupus nephritis (14 %) [13]. On the other hand, renal biopsy registry of china uncovered different trends. In that population 34.5 % of cases were diagnosed as IgA nephropathy followed by MCD (12.4 %) in recent years, according to Czech registry of renal biopsies [14]. Another study from Chinese population showed an even higher frequency of IgA nephropathy (40 %) [15]. In Romania, membrano-proliferative GN was found to be the changing dominant histologic pattern comprising of 29 % cases of primary GN [16].

In our population definitive trends cannot be reliably determined as there is lack of renal biopsy registry in our country. A few studies conducted so far showed differing patterns. A study involving 316 patients with nephrotic syndrome showed FSGS as the most common histologic pattern (39 %) followed by membranous nephropathy (26 %) [17]. Same trend was found in another study conducted in Karachi involving 60 cases of renal dysfunction with FSGS as the most common histologic pattern [18]. On the other hand study, Rabbani et al. found membrano-proliferative GN to be at the top of the list (28 %) followed by membranous GN (19 %) [19]. A study involving population from another part of the country found that MCD was the most common diagnosis on histology comprised of 40 % of cases [20]. We found a higher frequency of membranous GN. These conflicting patterns may represent different set of population characteristics in these studies. The studies with FSGS as the dominant pattern mostly represent rural population. On the other hand our study involved patients from urban parts of the country. Another explanation could be changing histologic patterns over a period of time as seen in other parts of the world.

Membranous GN is the term applied when renal biopsy shows diffuse thickening of glomerular basement membrane in the absence of significant hypercellularity [21, 22]. On Immunoflourescence studies IgG and C3c are usually demonstrated. In our study apart from IgG and C3c, significant number of cases also showed deposition of IgM and C1q. While most cases of membranous GN are idiopathic, in endemic areas like our country symptomatic Hepatitis B carrier state may be considered as a significant associated factor for the occurrence of membranous GN and its prevalence should be sought in populations at risk. Antibodies against M-type phospholipase A2 receptor (PLA2R) are serological markers of disease activity in patients with idiopathic membranous nephropathy. Some authors proposed that assessment of PLA2R antigen in biopsy specimens is a sensitive marker for the diagnosis of PLA2R-related MN [23]. We did not perform PLA2R antigen testing in our biopsy specimens.

In our study FSGS was the second most common pattern accounting for 18 % of cases of nephrotic syndrome. This represents a significant bulk of renal pathology in our population. An association of HIV infection with FSGS has been well established in western population, therefore HIV status should be determined in these cases.

One of the major limitations of our study was that all cases were of a single institution which could be a major source of bias, however as this is one of the largest tertiary care center in the province, therefore the patients which were referred to the nephrologist represent a major part of population including urban and rural parts of the province.

## Conclusion

In conclusion membranous GN is the most common histologic pattern seen in nephrotic syndrome followed by FSGS in our population, therefore we suggest that underlying risk factors like HBV and HIV infection should be determined in patients presenting with idiopathic nephrotic syndrome. More over there is a need for renal biopsy registry to determine evolving trends of nephrotic syndrome in our population.

## Consent

Written informed consent was obtained from the patients for publication of the data. Ethics committee of Liaquat National hospital approved the study.
